# Novel polymorphisms in *PDLIM3* and *PDLIM5* gene encoding *Z*‐line proteins increase risk of idiopathic dilated cardiomyopathy

**DOI:** 10.1111/jcmm.14607

**Published:** 2019-08-19

**Authors:** Dongfei Wang, Juan Fang, Jialan Lv, Zhicheng Pan, Xiang Yin, Hongqiang Cheng, Xiaogang Guo

**Affiliations:** ^1^ Department of Cardiology The First Affiliated Hospital, Zhejiang University School of Medicine Hangzhou China; ^2^ Department of Pathology and Pathophysiology Zhejiang University School of Medicine Hangzhou China

**Keywords:** idiopathic dilated cardiomyopathy, *PDLIM3*, *PDLIM5*, polymorphism

## Abstract

Idiopathic dilated cardiomyopathy (IDCM), characterized by ventricular dilation and impaired systolic function, is a primary cardiomyopathy resulting in heart failure. During heart contraction, the *Z*‐line is responsible for transmitting force between sarcomeres and is also a hot spot for muscle cell signalling. Mutations in *Z*‐line proteins have been linked to cardiomyopathies in both humans and mice. Actinin‐associated LIM protein (ALP) and enigma homolog protein (ENH), encoded by *PDLIM3* and *PDLIM5*, are components of the muscle cytoskeleton and localize to the *Z*‐line. A *PDLIM3* or *PDLIM5* deficiency in mice leads to dilated cardiomyopathy. Since *PDLIM3* and *PDLIM5* are candidate IDCM susceptibility genes, the current study aims to investigate whether polymorphisms within *PDLIM3* and *PDLIM5* could be correlated with IDCM. We designed a case‐control study, and exons of the *PDLIM3* and *PDLIM5* were amplified by polymerase chain reactions in 111 IDCM patients and 137 healthy controls. We found that five synonymous polymorphisms had statistical distribution differences between IDCM patients and controls, including rs4861669, rs4862543, c.731 + 131 T > G, c.1789‐3 C > T and rs7690296, according to genotype and allele distribution. Haplotype G‐C‐C‐C and A‐T‐C‐T (rs2306705, rs10866276, rs12644280 and rs4635850 synthesized) were regarded as risk factors for IDCM patients when compared with carriers of other haplotypes (all *P* < .05). Furthermore, IDCM patients with two novel polymorphisms (c.731 + 131 T > G and c.1789‐3 C > T) had lower systolic blood pressure. In conclusion, these five synonymous polymorphisms might constitute a genetic background that increases the risk of the development of IDCM in the Chinese Han population.

## 1 INTRODUCTION

Dilated cardiomyopathy (DCM) is a structural heart disease, characterized by ventricular dilation and impaired systolic function, in the absence of hypertension, coronary artery disease or valvular abnormalities,^1^ with an estimated prevalence of up to 1:2500.^2^ DCM is the third most frequent cause of heart failure and the most common condition requiring heart transplantation.^3^ Accumulating evidence has shown that the pathogenesis of DCM is associated with various factors, including virus‐mediated myocardial infection, autoimmune disease, toxic and metabolic damage, tachycardia, and genetic history.^2^ World Health Organization/International Society and Federation of Cardiology Task Force classifies primary DCM as idiopathic dilated cardiomyopathy (IDCM) with unknown causes differently from secondary DCM, such as hypertensive and ischaemic cardiomyopathy.^4^ Various genetic polymorphisms have been shown to be associated with increased risk of developing DCM.^5^ In most cases, these genes are involved in the encoding of myocardial skeleton, nuclear membrane, sarcomere, mitochondrial proteins and the calcium homeostasis regulating protein.^6^ The *Z*‐line, constituents of the myocyte cytoskeleton, defines the lateral boundaries of the sarcomere, provides an anchoring site for actin, titin and nebulin filaments, and mediates the transmission of tension between sarcomeres during contraction.^7^ Mutations in *Z*‐line proteins have been proven to be associated with cardiomyopathy or myofibrillar myopathy in humans.^8^ Actinin‐associated LIM protein (ALP) and enigma homolog protein (ENH), encoded by *PDLIM3* and *PDLIM5*, belong to the PDZ‐LIM protein family.^9^ Functionally, these proteins bind to α‐actinin at the *Z*‐line through their N‐terminal PDZ domains and bind to other proteins through their C‐terminal LIM domains.^9^

Actinin‐associated LIM protein, highly expressed in skeletal and cardiac muscle, has been suggested to play a pivotal role in myocyte stability, force generation and transmission, signal transduction, and mechanical signalling, especially in growth and remodelling processes.^9^ As for ENH, cardiac‐specific *PDLIM5* knockout mice developed DCM.^10^ We hypothesized that ALP and ENH, like its homologue Cypher, had an important role in the formation and maintenance of the normal *Z*‐line in cardiac muscle and are associated with human normal cardiac function. We hypothesized that the single nucleotide polymorphisms (SNPs) in *PDLIM3* and *PDLIM5* might be associated with susceptibility to IDCM. In order to confirm our hypothesis, we conducted this case‐control study, tested the candidate genes and analysed their association with IDCM, in the Chinese Han population.

## 2 MATERIALS AND METHODS

### 2.1 Study subjects

Between 2011 and 2018, 111 patients diagnosed with IDCM were enrolled in our study from the First Affiliated Hospital, School of Medicine, Zhejiang University. The clinical diagnosis of IDCM was based on the patient's history, physical examination, electrocardiogram, echocardiogram and coronary angiography, according to the revised criteria established in 1995 by the World Health Organization.^4^ All patients underwent standard transthoracic echocardiography.^11^ IDCM was defined as myocardiopathy with systolic dysfunction (left ventricular ejection fraction, LVEF < 45%) with or without left ventricular dilation, in the absence of heavy drinking, nutritional deficiency, hypocalcaemia, hypo‐ and hyper‐thyroidism, diabetes mellitus, autoimmune disease, hypertension, coronary artery disease or inflammatory DCM. Patients with a family history of DCM were also excluded.^12^ In addition, 137 healthy and unrelated controls were enrolled between 2017 and 2019. Each control was a healthy volunteer, defined as an asymptomatic individual without reduced left ventricular systolic function of left ventricular dilatation, who was normal based on detailed physical examination. All patients and controls were Han ethnicity from the same region in Zhejiang, China. To test the relationships between the SNPs and various clinical characteristics of IDCM, cases were stratified based on nine characteristics of patients with IDCM (Table 1). The study was approved by the ethics committee of the First Affiliated Hospital, School of Medicine, Zhejiang University, and informed written consent to perform genetic analysis was obtained from patients and controls, according to the second Helsinki Declaration.

**Table 1:** Baseline characteristics of IDCM patients

Parameter/characteristic	IDCM (n = 111)	NC (n = 137)	*P*
Age (y)	57.0 (48.0‐62.0)	53.0 (46.0‐60.5)	.11
Male	77 (69.4%)	90 (65.7%)	.59
NYHA classification ≥ 3	68 (61.3%)	NA	
LVEF (%) ( mean ± SD)	31.5 ± 8.7	NA	
LVEDD (cm) ( mean ± SD)	6.8 ± 0.9	NA	
SBP (mmHg) ( mean ± SD)	118.0 ± 17.0	NA	
ICD implantation	45 (40.5%)	None	
ACEI/ARB	52 (46.8%)	None	
Beta blocker	86 (77.5%)	None	

Note

Age at diagnosis is expressed as median (25th–75th percentile); all the other data are expressed as mean ± standard deviation (SD) for normally distributed parametric variables and percentage for categorical variables.

Abbreviations: ACEI/ARB, angiotensin‐converting enzyme inhibitor/angiotensin receptor blocker; BNP, brain natriuretic peptide; DBP, diastolic blood pressure; ICD, implantable cardioverter‐defibrillator; IDCM, idiopathic dilated cardiomyopathy; LBBB, left ventricular brunch block; LVEDD, left ventricular end‐diastolic dimensions; LVEF, left ventricular ejection fraction; NYHA, New York Heart Association; SBP, systolic blood pressure.

### 2.2 Genotyping

Genomic DNA of each individual was extracted from 200 µL EDTA‐anticoagulated peripheral blood samples by a DNA isolation kit from TIANamp Blood DNA Kit (TIANGEN) according to the manufacturer's instructions. The nucleotide sequences of the *PDLIM3* (NM_006457.4) and *PDLIM5* (NM_014476.5) genes were obtained from GenBank. Polymerase chain reaction (PCR) primers were designed by the Primer Premier 5.0 software and synthesized by Shanghai Sangon Biological Co., Ltd. The primers used in the genotyping analysis are listed in Table S1. PCR amplification was carried out to determine the exonic sequences of *PDLIM3* and *PDLIM5,* and the amplification products were purified. All gene exon sequences underwent sequencing in both the positive and negative directions followed by analysis using the DNAMAN software. The results were compared with the standard template sequences using NCBI BLAST and the CHROMAS software, to identify the gene mutation loci (sequencing was accomplished by China Hangzhou Tsingke Biological Testing Company).

### 2.3 Statistical analysis

Statistical analyses were performed with SPSS 19.0.0 (SPSS Inc). Continuous variables were reported as mean ± SD; categorical variables were summarized in terms of number and percentages. The chi‐square test was utilized to compare the difference in gender between IDCM patients and healthy controls, while the non‐parametric test was performed to determine any distinctions in age. Deviation from Hardy‐Weinberg equilibrium (HWE) for each SNP in the control group was assessed with a chi‐square goodness of fit test. Genotypic association tests in a case‐control pattern, assuming dominant, recessive, additive, over dominant and codominant genetic models, were performed using the Pearson chi‐square test. If expected frequencies in the cells of 2 × 2 tables were <5, Fisher's exact test was used. Odds ratio (OR) and respective 95% confidence intervals (CIs) were determined to assess the influence of any difference between genotypes, alleles and haplotypes. Correlation between variables and two new SNPs was determined using a non‐parametric test. Linkage disequilibrium (LD) among the SNPs and haplotype analysis were used by SHESIS software (Available online: http://analysis.bio-x.cn/myAnalysis.php). All reported *P* values were 2‐tailed, and statistical significance was set at *P* < .05.

## 3 RESULTS

### 3.1 Characteristics of participants

There were 248 unrelated participants, including 111 IDCM cases (77 men and 34 women, median age = 57.0 years) and 137 controls (90 men and 47 women, median age = 53.0 years). The baseline clinical characteristics of all participants are summarized in Table 1. In the IDCM group, the LVEF was 31.5 ± 8.7% and the left ventricular end‐diastolic dimensions (LVEDD) were 6.8 ± 0.9 cm on average. Of those, 45 underwent ICD implantation.

### 3.2 Identification of polymorphisms

Eight exons for *PDLIM3‐a* corresponding to *PDLIM3‐b‐d,* and 16 exons for *PDLIM5‐a* corresponding to isoforms *PDLIM5‐b‐I,* were screened. This screening strategy was based on high and specific expression of these isoforms in cardiac muscle, as well as mapping of the PDZ and LIM domains, within these isoforms. In the IDCM group, we found two new SNPs, the c.731 + 131 T > G and c.1789‐3 C > T (Figure S1). Moreover, these two new SNPs were not present in the 137 controls. In addition, we also identified nine SNPs already described as follows: rs4861669, rs4862543, rs2306705, rs10866276, rs12644280, rs4635850, rs7690296, rs2280003 and rs10031423 (Figure S1). Due to the A > G transversion at chr4:95 561 459 (c.1141A > G) in exon 9, rs7690296 led to the substitution of alanine for normal threonine at residue 84 (Thr84Ala). ExAC database search revealed that south Asian population frequencies for the 1141A > G allele were 0.6057 (52757/121090). However, in silico functional analyses showed that this variant is predicted to be irrelevant for *PDLIM5* (PolyPhen 0: benign, SIFT: tolerated and MutationTaster: polymorphism).

### 3.3 Association analysis of genotype and haplotypes of SNPs with the IDCM susceptibility

Because some participants' genes were failed to be sequenced, the total number of genotypes for some SNPs was less than 248. The frequency of each genotype in controls was consistent with the HWE hypothesis (Table 2). Table 3 shows the alleles and genotype frequencies of 11 SNPs, and the corresponding statistical analysis results. As shown in Table 3, significant differences in genotypes and allele frequencies were found at rs4861669, rs4862543, rs7690296 and two new SNPs, indicating that these polymorphisms may play an important role in the pathogenesis of IDCM. Our results showed that the minor allele of the two novel SNPs (c.731 + 131 T > G, OR = 62.07, 95% CI = 8.46‐455.22, *P* < .01; c.1789‐3 C > T, OR = 99.28, 95% CI = 13.22‐745.49, *P* < .01) was significantly related to IDCM. Since the minor allele was considered a relevant factor, we analysed the frequency in the dominant genetic model for both c.731 + 131 T > G and c.1789‐3 C > T. The results showed that the heterozygote genotype combined mutant genotype might also be related to IDCM (c.731 + 131 T > G, OR = 0.01, 95% CI = 0.00‐0.09, *P* < .01; c.1789‐3 C > T, OR = 0.01, 95% CI = 0.00‐0.08, *P* < .01). Additionally, the A allele frequency of rs4861669 (G > A) was strongly related to IDCM (OR = 1.71, 95% CI = 1.08‐2.69, *P* < .05). This indicated that the A allele of rs4861669 was associated with increased IDCM risk by 71%, compared with the G allele. Meanwhile, significant associations with IDCM were noted for rs4861669 in the dominant model (OR = 4.71, 95% CI = 1.02‐21.71, *P* = .03) and additive model (OR = 5.23, 95% CI = 1.12‐24.35, *P* = .02). The rs4862543 polymorphism was significantly associated with a predisposition to IDCM under the dominant, recessive, additive and codominant models across the whole IDCM population (Table 3). Nevertheless, no associated relationship could be found between rs2306705, rs10866276, rs12644280, rs4635850, rs2280003, and rs10031423 and the susceptibility to IDCM in codominant, dominant, recessive, over dominant or additive genetic model (all *P* > .05).

**Table 2:** Genomic characteristics

dbSNP ID	Genomic location	DNA changes	MAF in GnomAD	Alteration region	aa change	HWE
*PDLIM3*						
rs4861669	chr4:186444704	g.12063G > A	0.29	intron	–	.20
rs4862543	chr4:186444698	g.12069T > G	0.22	intron	–	.25
rs2306705	chr4:186427841	g.28926A > G	0.19	intron	–	.23
rs10866276	chr4:186423677	g.33090C > T	0.21	intron	–	.55
rs12644280	chr4:186423655	g.33112C > T	0.16	intron	–	.94
rs4635850	chr4:186423637	c.906C > T	0.21	CDS	–	.63
*PDLIM5*						
c.731 + 131 T > G	chr4:95529460	g.156424T > G	–	intron	–	1.00
rs7690296	chr4:95561459	c.1141A > G	0.44	CDS	T381A	.59
rs2280003	chr4:95575601	g.202565C > T	0.30	intron	–	.45
rs10031423	chr4:95583827	g.210791C > T	0.46	intron	–	.59
c.1789‐3 C > T	chr4:95585126	g.212090C > T	–	intron	–	1.00

Note

HWE (*P*) represents *P* value from Hardy‐Weinberg equilibrium test in the control group. T381A represents an alanine replacing for the normal threonine at residue 84.

Abbreviations: aa change, amino acid change; CDS, coding sequence; dbSNP, the single nucleotide polymorphism database; DNA, deoxyribonucleic acid; MAF, minor allele frequency; UTR, untranslated region.

**Table 3:** Allele and genotype distributions of *PDLIM3* and *PDLIM5* genetic polymorphisms in Han Chinese IDCM patients and control subjects

Gene	*PDLIM3*					
dbSNP ID	Model	Contrast	IDCM	Control	OR (95% CI)	*P*
rs4861669	Dominant	GA + AA/GG	107/2	125/11	4.71 (1.02, 21.71)	.03
	Recessive	AA/GA + GG	76/33	80/56	1.61 (0.95, 2.75)	.08
	Additive	AA/GG	76/2	80/11	5.23 (1.12, 24.35)	.02
	Over dominant	GG + AA/GA	78/31	91/45	1.24 (0.72, 2.15)	.43
	Codominant	AA	76	80	1.00	.05
		GA	31	45	0.73 (0.42, 1.26)	
		GG	2	11	0.19 (0.04, 0.89)	
	Allelic	A/G	183/35	205/67	1.71 (1.08, 2.69)	.02
rs4862543	Dominant	GT + GG/TT	106/2	57/79	0.01 (0.00, 0.06)	.00
	Recessive	GG/GT + TT	76/32	11/125	0.04 (0.02, 0.08)	.00
	Additive	GG/TT	76/2	11/79	0.00 (0.00, 0.02)	.00
	Over dominant	TT + GG/GT	78/30	90/46	0.75 (0.43, 1.31)	.31
	Codominant	GG	76	11	1.00	.00
		GT	30	46	0.09 (0.04, 0.21)	
		TT	2	79	0.00 (0.00, 0.02)	
	Allelic	G/T	182/34	68/204	16.06 (10.16, 25.38)	.00
rs2306705	Dominant	GA + GG/AA	106/5	127/10	0.60 (0.20, 1.81)	.36
	Recessive	GG/GA + AA	70/41	83/54	0.90 (0.54, 1.51)	.69
	Additive	GG/AA	70/5	83/10	0.59 (0.19, 1.82)	.36
	Over dominant	AA + GG/GA	75/36	93/44	1.02 (0.59, 1.73)	.96
	Codominant	GG	70	83	1.00	.63
		GA	36	44	0.97 (0.56, 1.67)	
		AA	5	10	0.59 (0.19, 1.82)	
	Allelic	G/A	176/46	210/64	1.17 (0.76, 1.79)	.48
rs10866276	Dominant	CT + TT/CC	85/2	129/8	0.38 (0.08, 1.83)	.36
	Recessive	TT/CT + CC	49/38	84/53	1.23 (0.71, 2.12)	.46
	Additive	TT/CC	49/2	84/8	0.43 (0.09, 2.10)	.47
	Over dominant	CC + TT/CT	51/36	92/45	1.44 (0.83, 2.52)	.20
	Codominant	TT	49	84	1.00	.25
		CT	36	45	1.37 (0.78, 2.41)	
		CC	2	8	0.43 (0.09, 2.10)	
	Allelic	T/C	134/40	213/61	0.96 (0.61, 1.51)	.86
rs12644280	Dominant	CT + TT/CC	40/47	72/65	1.30 (0.76, 2.23)	.34
	Recessive	TT/CT + CC	5/82	13/124	1.72 (0.59, 5.01)	.32
	Additive	TT/CC	5/47	13/65	1.88 (0.63, 5.63)	.25
	Over dominant	CC + TT/CT	52/35	78/59	0.89 (0.52, 1.54)	.68
	Codominant	TT	5	13	1.00	.48
		CT	35	59	1.54 (0.51, 4.69)	
		CC	47	65	1.88 (0.63, 5.63)	
	Allelic	T/C	45/129	85/189	0.78 (0.51, 1.19)	.24
rs4635850	Dominant	CT + TT/CC	84/3	129/8	0.58 (0.15, 2.23)	.62
	Recessive	TT/CT + CC	49/38	83/54	1.19 (0.69, 2.06)	.53
	Additive	TT/CC	49/3	83/8	0.64 (0.16, 2.51)	.74
	Over dominant	CC + TT/CT	52/35	91/46	1.33 (0.76, 2.32)	.31
	Codominant	TT	49	83	1.00	.49
		CT	35	46	1.29 (0.73, 2.27)	
		CC	3	8	0.64 (0.16, 2.51)	
	Allelic	T/C	133/41	212/62	0.95 (0.61, 1.49)	.82

Gene	*PDLIM5*					
dbSNP ID	Model	Contrast	IDCM	Control	OR (95% CI)	*P*
c.731 + 131 T > G	Dominant	GT + GG/TT	41/70	0/137	0.01 (0.00, 0.09)	.00
	Codominant	GG	0	0	–	–
		GT	41	0	–	
		TT	70	137	–	
	Allelic	G/T	41/181	0/274	62.07 (8.46, 455.22)	.00
rs7690296	Dominant	GA + GG/AA	61/46	84/53	1.20 (0.71, 2.00)	.50
	Recessive	GG/GA + AA	13/94	22/115	1.38 (0.66, 2.89)	.39
	Additive	GG/AA	13/46	22/53	1.47 (0.67, 3.24)	.34
	Over dominant	AA + GG/GA	59/48	75/62	0.98 (0.59, 1.64)	.95
	Codominant	GG	13	22	1.00	.63
		GA	48	62	1.31 (0.60, 2.87)	
		AA	46	53	1.47 (0.67, 3.24)	
	Allelic	G/A	74/140	168/106	0.33 (0.23, 0.48)	.00
rs2280003	Dominant	CT + TT/CC	61/50	73/64	0.94 (0.57, 1.55)	.79
	Recessive	TT/CT + CC	10/101	11/126	0.88 (0.36, 2.16)	.78
	Additive	TT/CC	10/50	11/64	0.86 (0.34, 2.18)	.75
	Over dominant	CC + TT/CT	60/51	75/62	1.03 (0.62, 1.70)	.91
	Codominant	TT	10	11	1.00	.95
		CT	51	62	0.91 (0.36, 2.30)	
		CC	50	64	0.86 (0.34, 2.18)	
	Allelic	T/C	71/151	84/190	1.06 (0.73, 1.56)	.75
rs10031423	Dominant	CT + TT/CC	53/27	87/50	0.89 (0.50, 1.58)	.68
	Recessive	TT/CT + CC	13/67	19/118	0.83 (0.39, 1.79)	.63
	Additive	TT/CC	13/27	19/50	0.79 (0.34, 1.84)	.58
	Over dominant	CC + TT/CT	40/40	69/68	1.02 (0.59, 1.76)	.96
	Codominant	TT	13	19	1.00	.86
		CT	40	68	0.86 (0.38, 1.93)	
		CC	27	50	0.79 (0.34, 1.84)	
	Allelic	T/C	66/94	106/168	1.11 (0.75, 1.66)	.60
c.1789‐3 C > T	Dominant	CT + TT/CC	25/22	0/137	0.01 (0.00, 0.05)	.00
	Codominant	TT	0	0	–	–
		CT	25	0	–	
		CC	22	137	–	
	Allelic	T/C	25/69	0/274	99.28 (13.22, 745.49)	.00

Abbreviations: CI, confidence interval; dbSNP, the single nucleotide polymorphism database; IDCM, idiopathic dilated cardiomyopathy; OR, odds ratio.

**Figure 1:**
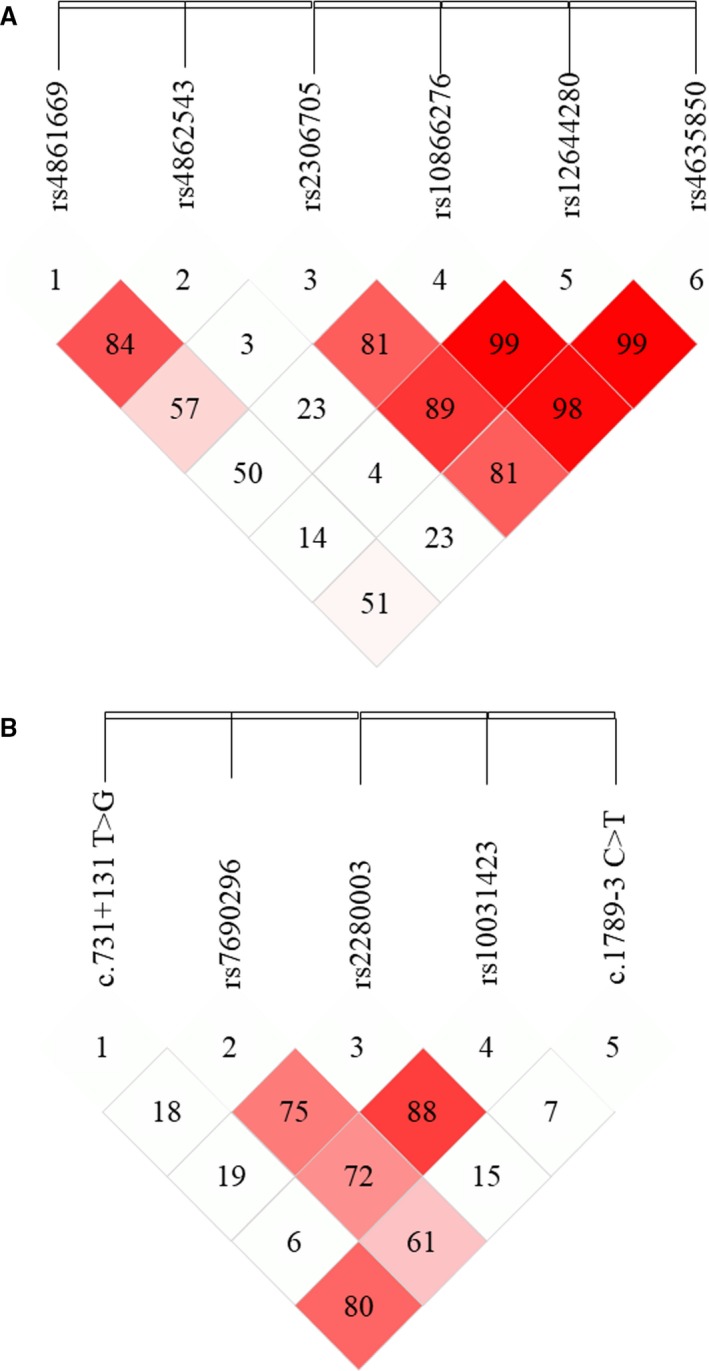
Linkage disequilibrium map. A, six SNPs within *PDLIM3* and (B) five SNPs within *PDLIM5*. The *D*′ values were shown inside each diamond (stronger correlations between these SNPs are noted by red colour). Map was drawn based on the genotype data of all case and control samples using SHESIS. SNP, single nucleotide polymorphism

We estimated LD among the six SNPs in *PDLIM3* and five SNPs in *PDLIM5* by using SHESIS software. As displayed in Figure 1, the SNP pairs rs2306705/rs10866276/rs12644280/rs4635850 in *PDLIM3* exhibited strong linkage disequilibrium (*D*′ > 0.75). Subsequently, a total of 16 haplotypes were obtained after randomly combining the four SNPs in *PDLIM3* based on SHESIS software; however, five haplotypes were finally excluded for their low frequency in the studied population (each lower than 3%). Partial haplotype frequencies of the *PDLIM3* gene, in patients and controls, are summarized in Table 4. It was finally indicated, by haplotype analyses, that haplotypes G‐C‐C‐C and A‐T‐C‐T (rs2306705, rs10866276, rs12644280, and rs4635850 synthesized) were regarded as risk factors for IDCM patients, compared with carriers of other haplotypes (OR = 4.09, 95% CI = 1.27‐13.14; OR = 3.2, 95% CI = 1.00‐10.50, respectively).

**Table 4:** *PDLIM3* haplotype distribution and effect on IDCM

Haplotype	Frequency		*χ* ^2^‐test	OR (95% CI)	*P*
	IDCM	Control			
GTTT	43.6 (0.25)	82.2 (0.30)	1.10	0.79 (0.52, 1.22)	.79
GTCT	78.7 (0.45)	123.7 (0.45)	0.03	1.03 (0.70, 1.52)	.86
GCCC	10.0 (0.06)	4.1 (0.02)	6.48	4.09 (1.27, 13.14)	.01
ATCT	8.4 (0.05)	4.3 (0.02)	4.2	3.2 (1.00, 10.50)	.04
ACCC	28.9 (0.17)	56.9 (0.21)	1.04	0.77 (0.47, 1.27)	.31

Note

Loci chosen for haplotype analysis: *PDLIM3*:rs2306705, rs10866276, rs12644280, rs4635850.

Abbreviations: CI, confidence interval; IDCM = idiopathic dilated cardiomyopathy; OR, odds ratio; χ^2^‐test = chi‐square test.

### 3.4 Two new SNPs within PDLIM5 were significantly associated with systolic blood pressure in patients with IDCM

Subgroup analysis was performed to evaluate the potential relationship between the genotypes of two new SNPs within *PDLIM5,* and clinical features such as SBP, LVEDD and LVEF (Tables 3, 4 and 5). The results of the subgroup analysis showed that the presence of the TT genotype for c.731 + 131 T > G and CC genotype for c.1789‐3 C > T was associated with lower SBP (TT, *P* = .00; CC, *P* = .01; dominant model for both; Table 5). However, no significant relationship was found between LVEDD and LVEF and the genotype of the two new SNPs, in IDCM subjects.

**Table 5:** Association of 2 novel *PDLIM5* gene polymorphisms with clinical characteristics in the IDCM patients

Characteristics	c.731 + 131 T > G			c.1789‐3 C > T		
	Genotype	Mean ± SD	*P* value	Genotype	Mean ± SD	*P* value
SBP	GT + GG	111.5 ± 13.4	.00	CT + TT	111.7 ± 13.5	.01
	TT	121.8 ± 17.7		CC	126.5 ± 19.8	
LVEF	GT + GG	30.8 ± 8.1	.56	CT + TT	30.8 ± 6.6	.77
	TT	31.9 ± 9.0		CC	30.9 ± 8.9	
LVEDD	GT + GG	6.9 ± 1.0	.17	CT + TT	6.8 ± 1.2	.39
	TT	6.7 ± 0.8		CC	6.6 ± 0.9	

Abbreviations: LVEDD, left ventricular end‐diastolic dimensions; LVEF, left ventricular ejection fraction; IDCM, idiopathic dilated cardiomyopathy; SBP, systolic blood pressure.

## 4 DISCUSSION

IDCM is estimated to be of genetic origin in up to 50% of all cases.^13^ To date, mutations in many of *Z*‐line‐associated proteins have been proven to be linked to cardiomyopathy in both humans and transgenic mouse models,^8^, ^14^ suggesting that *Z*‐line proteins play a pivotal role in the pathogenesis of cardiomyopathy. One such protein is Cypher, a PDZ and LIM domain‐containing protein, that was first cloned in 1999.^15^ *Cypher*‐deficient mice display premature lethality with severe DCM and disorganized *Z*‐lines.^16^ Furthermore, many mutations in the human *Cypher/ZASP* gene have been identified in patients with DCM.^8^ Compared to Cypher, less is known about the role of the other two PDZ‐LIM proteins, ALP and ENH, in the heart. Functionally, these proteins participate in the formation of sarcomeric complexes at the *Z*‐line and interact with various proteins through their LIM domains, particularly with signalling factors.^9^ ALP and ENH are essential for proper heart development and contractility in hearts, as a *PDLIM3* or *PDLIM5* deficiency in mice results in DCM.^9^, ^10^, ^17^ The important roles of ALP and ENH in heart function make them two promising, candidate genes for cardiomyopathy. The association of polymorphisms of *PDLIM3* and *PDLIM5* with susceptibility to IDCM, however, remains unexplored.

The present analysis of the data, from 111 IDCM patients and 137 controls, identified five SNPs in the *PDLIM3* and *PDLIM5* genes associated with IDCM. These two genes are considered in more detail below.

ALP is highly expressed in striated muscle (Xia, 1997).^18^ *PDLIM3* resides in chromosome 4q35.1, abnormal splicing of *PDLIM3* that is deleted in myotonic dystrophy type 1.^19^ *PDLIM3* is a conserved human gene: the EXaC exome aggregation consortium server (http://exac.broadinstitute.org/, accessed as of October 2018) estimates that the frequency of *PDLIM3* LOF variants is <1/10 000. A potentially pathogenic frameshift mutation (M60Tfs1X) of *PDLIM3* was reported in a patient with DCM.^13^ Meanwhile, in mice, ablation of *PDLIM3* results in primarily right ventricular dysmorphogenesis, a decrease in trabeculation and mild DCM.^17^ Moreover, Elisabeth and coworkers found that tight regulation of ALP is essential for a proper balance between maintenance of the extracellular matrix and the formation of fibrotic scars.^20^ Cardiac fibrosis is a prominent feature of several cardiomyopathies, which reduces cardiac contractility and electric conductivity.^21^ In addition, many disease‐related polymorphisms in non‐coding regions can affect the expression level of coding proteins by disrupting the transcription factor recognition sequence in related cell types.^22^

Within the *PDLIM3* gene, we found two SNPs (rs4861669 and rs4862543) associated with IDCM. Allele A of rs4861669 was closely related, with higher risk of IDCM (allele A: OR = 1.71, 95% CI = 1.08‐2.69). By using the chi‐square test, we found that a significantly increased risk of IDCM was associated with the AA/GA genotypes, compared with GG genotype, in the dominant model. A significantly increased risk of IDCM was also found to be associated with the AA genotype of rs4861669 in the additive model, compared with GG genotypes, which shows homozygote advantage. The same held true for rs4862543.

The *PDLIM5* gene is located at 4q22.3. *PDLIM5* encodes several splice variants, whose expression is tissue specific and temporally regulated.^9^ Alternative splicing plays an important role in heart development and in the development of cardiopathies.^23^ The splice variants of *PDLIM5* can be divided into two groups, long isoform (ENH1), containing the three LIM domains, and short isoforms, lacking the three LIM domains.^9^ Maturana and coworkers showed that ENH1 forms a complex with protein kinase D1 and the alpha1C subunit of cardiac L‐type voltage‐gated calcium channels in rat neonatal cardiomyocytes to regulate the activity of the channel.^24^ ENH1 is highly expressed in foetal and neonatal hearts, down‐regulated in adult hearts, but up‐regulated in pressure‐overload hypertrophy, while short isoforms appear to show the opposite pattern.^25^ ENH1 was originally found as protein kinase C (PKC)‐interacting protein and has an important role in heart development, by scaffolding PKCβ to the *Z*‐line.^26^ It is well‐established that the activation of PKC (particularly PKCα and PKCβ) and PKCε is an important signalling pathway in the development of heart diseases, including DCM.^27^ Indeed, a point mutation on *Cypher*'s LIM domains increases the binding affinity for PKC and is associated with DCM of patients carrying this mutation.^28^ Although no studies have yet reported the association between *PDLIM5* mutations and cardiomyopathy, *PDLIM5* gene mutations may actually lead to an increased susceptibility to diseases such as cancer, bipolar disorder, hypertension, alcohol dependence and schizophrenia.^29^, ^30^, ^31^

We identified five *PDLIM5* SNPs, including one missense SNP (rs7690296) and four synonymous SNPs (rs228003, rs10031423, c.731 + 131 T > G and c.1789‐3 C > T). Only rs7690296 and two novel SNPs (c.731 + 131 T > G and c.1789‐3 C > T) were associated with a risk of IDCM. The missense mutation at the rs7690296 locus replaces the amino acid Thr with Ala. SNPs with missense mutations can alter the structure and/or function of a gene. Furthermore, its allele frequencies were significantly associated with IDCM. One possible explanation is that a mutation at rs7690296 alters the amino acid sequence in a way that affects the function of the protein. The minor alleles of both two novel SNPs were significantly associated with higher IDCM risk. Moreover, under dominant model, two novel SNPs were also associated with a lower level of SBP, in IDCM patients. Further studies are still required to verify the significance of our results and to explore the role of these two novel SNPs in the pathophysiologic mechanisms of DCM. Also, the haplotype consisting of four highly linked SNPs implied that G‐C‐C‐C and A‐T‐C‐T carriers were more inclined to suffer from DCM than carriers of other haplotypes.

There are still some limitations to our research. The main limitations are the relatively small sample size of the study population and the lack of replication of this significant association in a second, independent cohort of IDCM patients. This is also a single‐centre study; thus, we cannot exclude the presence of selection bias in patient enrolment. Our study was limited to the genetic level, and functional studies are therefore required to explore the molecular mechanisms by which SNPs would affect the gene expression of *PDLIM3* and *PDLIM5*.

## 5 CONCLUSION

Our study demonstrates, for the first time, that polymorphisms in *PDLIM3* (rs4861669, rs4862543) and *PDLIM5* (rs1056772) were significantly associated with IDCM in Chinese Han patients. We also identified 2two novel SNPs (c.731 + 131 T > G and c.1789‐3 C > T) in the intron of the *PDLIM5* gene that may increase the risk of IDCM and affect SBP level in IDCM patients. This study is an important step towards establishing a link between genetic polymorphisms in *Z*‐line protein genes, *PDLIM3* and *PDLIM5*, and IDCM.

## CONFLICT OF INTEREST

The authors declare no conflicts of interest associated with the manuscript.

## AUTHORS' CONTRIBUTIONS

DFW, JF and JLL executed the experiments. DFW, HQC and XGG designed the study. JF, ZCP and XY performed data analysis. DFW and XGG wrote the manuscript while JF and JLL assisted with manuscript editing. The final version was approved by all authors.

## Data Availability

The data used to support the findings of this study are available from the corresponding author on request.
