# Supplementary material for: Novel polymorphisms in PDLIM3 and PDLIM5 gene encoding Z‐line proteins increase risk of idiopathic dilated cardiomyopathy
Source: J Cell Mol Med. 2019 Aug 19;23(10):7054–62. doi: 10.1111/jcmm.14607 (PMC6787498; doi:10.1111/jcmm.14607)
Supplement: Supplementary file 1 [file JCMM-23-7054-s001.docx]

**Supplemental table 1. Primer sequences used for amplification of exons in *PDLIM3* and *PDLIM5*.**

| Gene | PCR amplification primer | |
| --- | --- | --- |
| *PDLIM3* | Foward(5’→3’) | Reverse(5’→3’) |
| Exon1 | CGCGGGGACACTTAGAGC | CTGCGGACCCAGAGGATG |
| Exon2 | ACTCAAGATACTGCGGGATGTTGG | GGAAACGGCAGATTGGTG |
| Exon3 | TCTAGTCTGCCATACCCA | TAACCGTAGATTGGAGACAC |
| Exon4 | GTTTAGCGGATAGATGGT | TGACCTGGGATAGACATAC |
| Exon5 | AGTGAAACAGAGCCAAAA | ATTTGGTCCTTACCTGAT |
| Exon6 | AGTGGCTGTGCATAGAGT | GAGGCTGAGAAGTTTGAC |
| Exon7 | TCAGAACAGCGTCCCTTAT | AAATGAACTGTCGCCAAG |
| Exon8 | GCCCGGTCGTCTTTGATC | GCATTTGCCTCCCATTCC |
| *PDLIM5* |  |  |
| Exon1 | ATACATGAAGTGGAGGGAAGGA | GAATTCCCCAAATTAGGGACA |
| Exon2 | TACTGTTGCACAATAAAGAAATGG | GCCTTTTGATGGGGAAATAAC |
| Exon3 | TGAAACGATACATTCCAGTGATTC | GAGGGATACTTGCCAATAACCTA |
| Exon4 | AGTCACATCCATCCCATCACC | AGGAAGAAAGATTCAAGATTTGC |
| Exon5 | CTGCTGCCTTCCTCTGTCAAT | CTTGGTGGGCT |
| Exon6 | ACATCCCAGGTTCAAGCG | GACAGAGCCCCTCCATCA |
| Exon7 | TTGGTGCAGGTAATTGCTTAAG | TGTTCATCTTCCTTTTCTCACAAC |
| Exon8 | TTTGTGGTGGCAGAAGTG | TGGGCACCTGGTAACATA |
| Exon9 | TGCTACCCTTTCTCACTT | TCACATCAGACAGACGCT |
| Exon10 | TAAATGATGGCTTTGAC | CCAAGCAAAAC |
| Exon11 | TGTTCTGTTAGGCAAGGGTTG | CTGCTGCTCAAATGCTCTT |
| Exon12 | ACCTACATAGTCACAGCCTA | ACCCAACATTGATATTAAGT |
| Exon13 | ATCAATGCAGAGTCCCTC | CCAAAGACAACCTAACCC |
| Exon14 | TTGGCATTTGTAAAGAACAC | GAGAATCGCCTGAACCTG |
| Exon15 | CTCAAGCCAAATGTTATG | TTCCCAGTGTTAATCCAATA |
| Exon16 | GCATACTGGGTAAAATCC | TAATAAATGCCAAACCGT |

PCR=polymerase chain reaction.


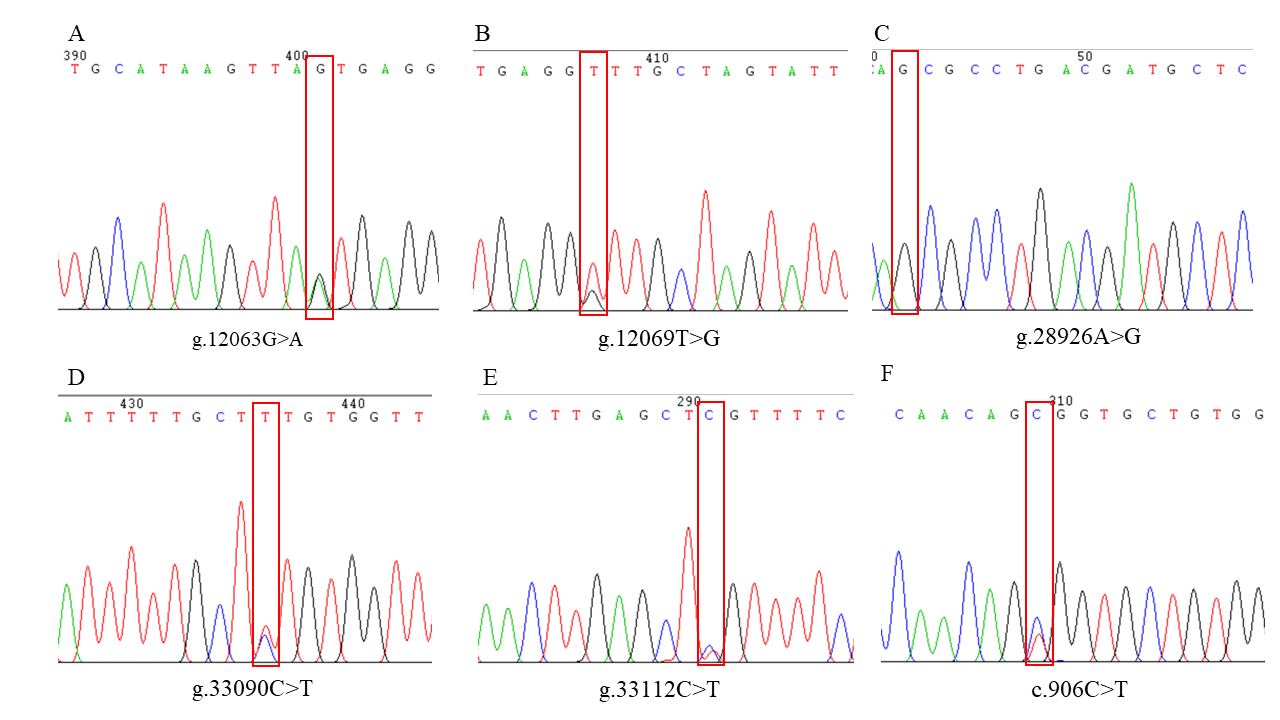


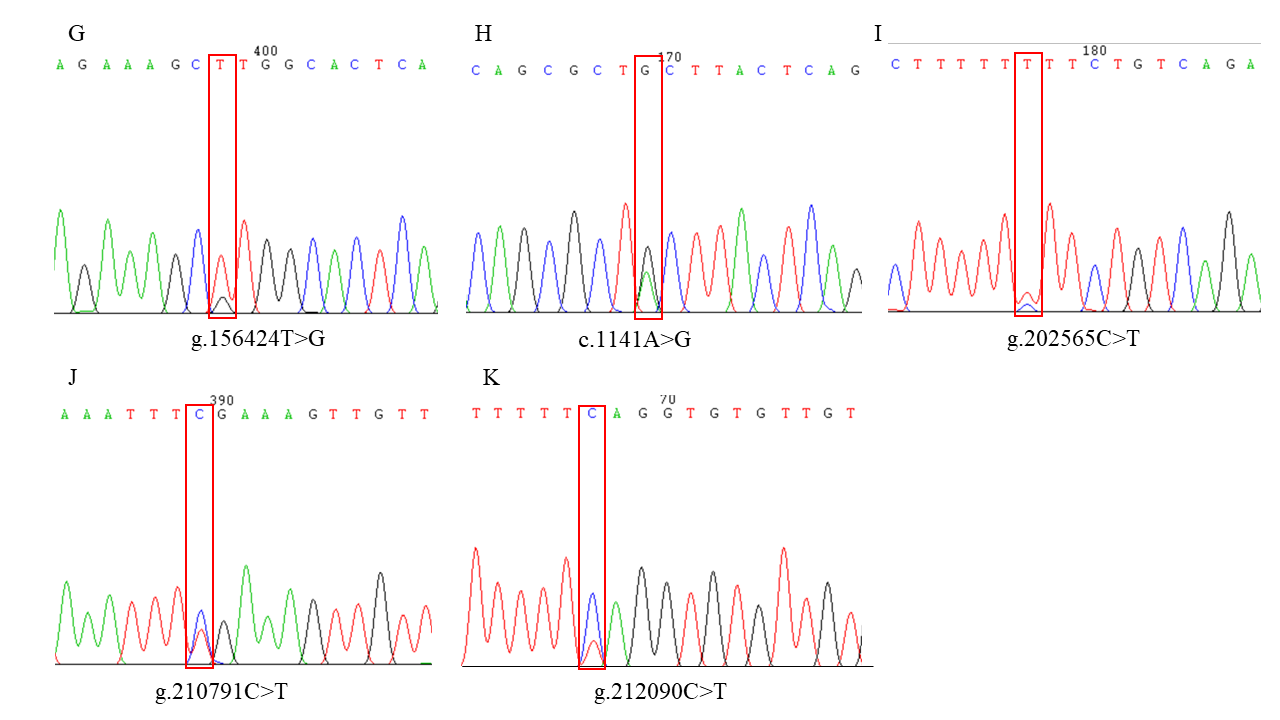


Supplemental figure 1. Sequences of *PDLIM3* and *PDLIM5*. Six polymorphisms in *PDLIM3* gene (A-F) and five in *PDLIM5* gene (G-K) in IDCM patients, including a missense mutation (c.1141 A>G, Thr381Ala).

IDCM=idiopathic dilated cardiomyopathy.
